# Community‐driven variations in snow algae color modulate snow albedo reduction

**DOI:** 10.1111/nph.70775

**Published:** 2025-11-25

**Authors:** Pablo Almela, James J. Elser, Anthony Zmuda, Thomas Niehaus, Trinity L. Hamilton

**Affiliations:** ^1^ Department of Plant and Microbial Biology University of Minnesota St Paul MN 55108 USA; ^2^ Flathead Lake Biological Station University of Montana Polson MT 59860 USA

**Keywords:** albedo, astaxanthin, community composition, phylogenetic analysis, pigment signature, red snow, snow algae, snow reflectance

## Abstract

- Snow algae blooms visibly alter snow color and surface energy balance, yet the biological basis of this variability remains unclear. We investigated how pigment composition and community structure shape the optical properties of snow algae blooms of distinct colors – red, orange, and green – co‐occurring within the same snowfield in Glacier National Park, USA.
- We measured the spectral reflectance, pigment composition (HPLC), and algal community composition (18S rRNA amplicon sequencing) of each bloom type to quantify how biological characteristics influence snow reflectance and radiative forcing.
- Astaxanthin dominated all blooms, while Chl*a* was most abundant in green blooms. Distinct algal taxa characterized each color, with *Sanguina* dominating red blooms and *Chloromonas* being more abundant in green and orange. Red blooms showed the lowest reflectance and highest radiative forcing (56 W m^−2^), exceeding that of green (21 W m^−2^) and orange blooms (25 W m^−2^), enhancing energy absorption into the snowpack and promoting localized melting of adjacent ice crystals.
- Our data indicate that bloom color reflects distinct community compositions, characterized by differences in dominant taxa and pigment pools, which together drive the radiative balance of snowfields. However, these relationships may not be universal, and color is best viewed as an emergent property shaped by multiple biological and environmental factors.

## Resumen

- Los *blooms* de algas de la nieve alteran el color y el balance energético de la superficie de la nieve, aunque la base biológica de esta variabilidad sigue siendo poco clara. Analizamos cómo la composición de pigmentos y la estructura de la comunidad determinan las propiedades ópticas de *blooms* de distinto color – rojo, naranja y verde – que coexisten en un mismo campo de nieve en el Parque Nacional de los Glacieres (EE.UU).
- Medimos la reflectancia espectral, la composición de pigmentos (HPLC) y la composición de la comunidad algal (secuenciación de amplicones 18S rRNA) para cuantificar cómo las características biológicas influyen en la reflectancia de la nieve y en el forzamiento radiativo.
- La astaxantina fue el pigmento dominante en todas los *blooms*, mientras que la clorofila‐a fue más abundante en las verdes. Cada color presentó taxones distintos, con *Sanguina* dominante en las rojas y *Chloromonas* más abundante en las verdes y naranjas. Las floraciones rojas mostraron la menor reflectancia y el mayor forzamiento radiativo (56 W m^−2^), superando al de las verdes (21 W m^−2^) y naranjas (25 W m^−2^), lo que incrementa la absorción de energía en la nieve y favorece la fusión localizada de los cristales de hielo.
- Nuestros resultados indican que el color refleja composiciones comunitarias distintas, definidas por diferencias en los taxones dominantes y la composición de pigmentos, que determinan el balance radiativo de la nieve. Sin embargo, estas relaciones pueden no ser universales y el color debe entenderse como una propiedad emergente moldeada por factores biológicos y ambientales.

## Introduction

The presence of light‐absorbing particles (LAPs) on the surface of snow, whether abiotic (e.g. mineral dust and black carbon) or biotic (e.g. snow algae), leads to a significant decrease in albedo and an increase in melting rates (e.g. Stibal *et al*., 2017; Skiles *et al*., 2018; Cook *et al*., 2020; Engstrom *et al*., 2022). These particles often co‐occur, amplifying their impact. For example, in the Alps, a combination of red snow algae and mineral dust reduced albedo by 41% (Di Mauro *et al*., 2024). On average, these reductions were estimated at 7.4% for snow algae alone, 35.3% for mineral dust, and 40.8% when both are present. Studies focusing exclusively on snow algae have estimated albedo reductions ranging from 13% to 20% on average (Thomas & Duval, 1995; Lutz *et al*., 2016; Gray *et al*., 2020), with reductions reaching up to 40% in maritime Antarctica (Khan *et al*., 2021). Increased melt by biotic LAPs accelerates the availability of liquid water necessary for algal growth, ensuring water availability in a highly illuminated but water‐limited environment (Dial *et al*., 2018) and creates a feedback loop where increased algal abundance further enhances melting (Ganey *et al*., 2017). With permanent and seasonal snow covering up to 35% of the Earth's surface (Hell *et al*., 2013), clear potential exists for widespread contributions of snow algae to snow melt.

Snow algae influence the spectral albedo of snow, particularly in the visible range (400–700 nm), by darkening the surface and increasing radiative forcing (Warren & Wiscombe, 1980). Their effect on snow reflectance is closely tied to pigment composition, which dictates snow coloration. However, the relationship between environmental conditions and pigment production, which can influence visible snow coloration at high cell concentrations, remains uncertain. While UV exposure and nutrient limitation are considered key regulators of secondary carotenoid synthesis, findings are inconsistent. Leya *et al*. (2009) observed that nitrogen starvation and high light levels in lab cultures induced carotenoid accumulation, turning algae reddish. By contrast, astaxanthin‐rich algae have been reported in nitrogen‐rich snow (Fujii *et al*., 2010), while other studies have found no direct correlation between carotenoid levels and meltwater nutrient concentrations (Müller *et al*., 1998). These divergent results suggest that pigment production in snow algae varies not only with environmental conditions but also between species, depending on their ecological strategies and metabolic pathways. For example, Nakashima *et al*. (2021) reported variations in pigment composition of different bloom colors on snow associated with algal species composition. This indicates that different algal species may respond uniquely to environmental factors, which could explain some of the variability in observed patterns of pigment composition and subsequent impacts on albedo.

Despite growing interest in snow algae and their quantifying their contribution to snow melt, few studies have simultaneously examined algal biomass, pigment composition, and radiative forcing while considering different snow algal colors (Gray *et al*., 2020; Khan *et al*., 2021; Halbach *et al*., 2022). This may be due to the rarity of observing abundant blooms of different colors in the same location at the same time. Because the response to environmental stressors may be species‐dependent, accounting for both the taxonomic composition of snow algae and their pigment composition (and thus bloom color) is essential for understanding and predicting biological contributions to albedo reduction.

In this study, we examined the community composition, biomass abundance, pigment composition, and radiative forcing of red, orange, and green snow algae blooms occurring concurrently in the same snowfield. The presence of different species and pigment pools under similar snow conditions allowed us to evaluate the impact of varying colors on the optical properties of an alpine snowfield. Additionally, we explored the potential of estimating algal biomass with differing carotenoid contents using spectral regions typically employed by high‐resolution satellites.

## Materials and Methods

### Field site description

**Fig. 1:**
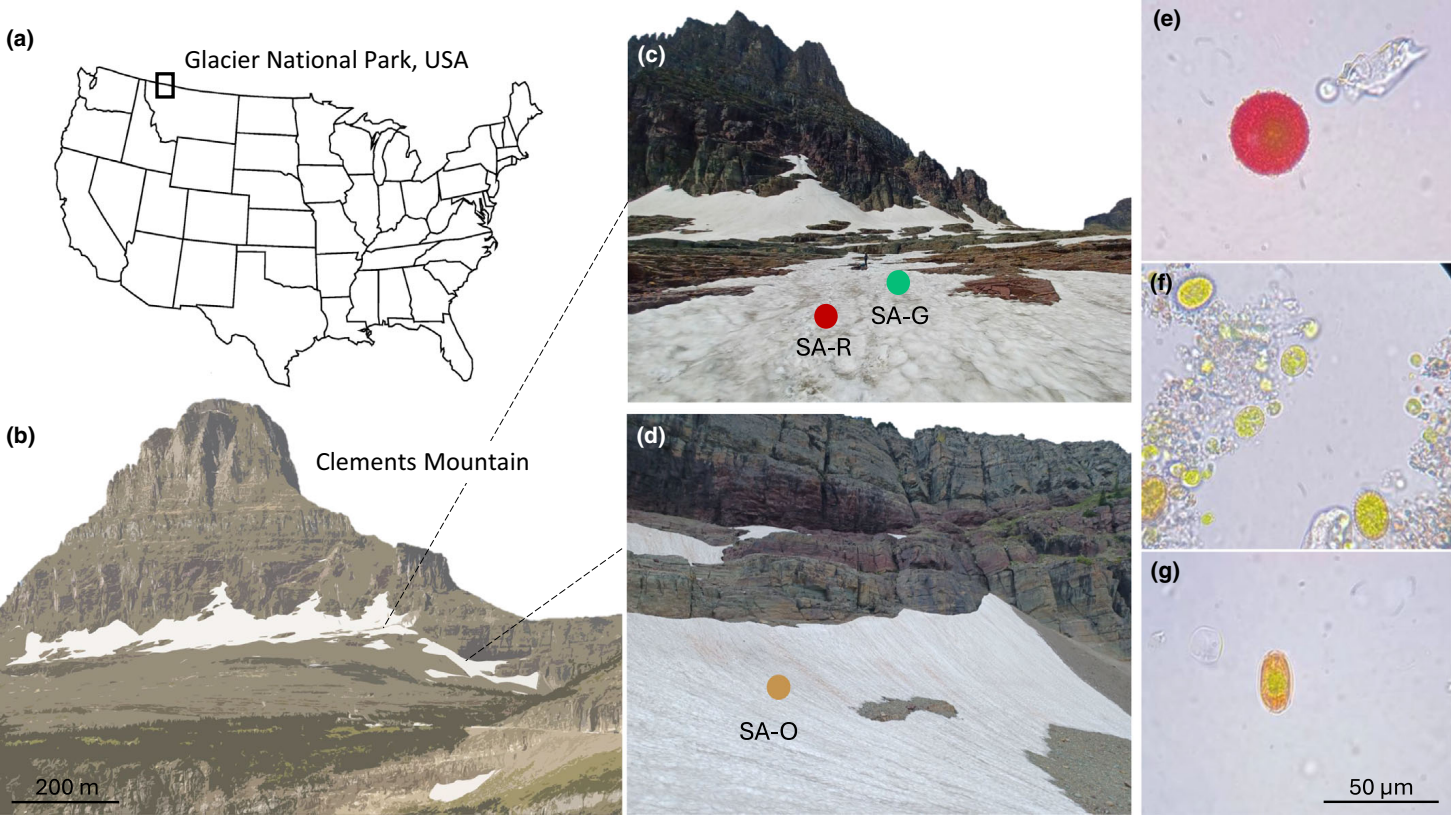
Sampling and measurement site locations. (a) Glacier National Park and (b) Clements Mountain, where red (SA‐R), green (SA‐G), and orange (SA‐O) snow algae blooms were studied. (c, d) The dots indicate the approximate locations where the reflectance of clean snow was measured. Photos show the dominant cell types in the different colored blooms, which likely correspond to *Sanguina nivaloides* (e), *Chloromonas alpina* (f), and *Chloromonas* sp. (g) according to the predominant 18S rRNA sequences obtained from those samples.

Glacier National Park (GNP), referred to as Ya·qawiswit̓xuki (‘the place where there is a lot of ice’) by the Kootenai tribe, is located in northwest Montana, USA (Fig. 1a). During the Little Ice Age, an estimated 146 glaciers were within the current boundaries of GNP. At the end of this period, *c*. 1850, there were *c*. 80 glaciers in what would eventually become the national park. Only 51 of these glaciers persisted until 2005 (Martin‐Mikle & Fagre, 2019). This ongoing glacial retreat makes GNP a site of great relevance for the study of climate change and cryosphere responses.

Sampling was carried out on August 9, 2024, between 13:00 h and 15:00 h, on a seasonal snowfield in an open area above timberline that extended across the northeast base of Clements Mountain (48°41′33″N, 113°44′10″W) at Logan Pass. The snowfield was divided into two sections by a moraine, with slopes ranging from 3° to 19°. The red and green algae blooms were found close to each other (*c*. 10 m apart), whereas the orange snow algae bloom was in a smaller section of the snowfield, *c*. 350 m away (Fig. 1b). Sampling was targeted (non‐random): samples were specifically collected from snowpack that appeared red, orange, or green. These areas of snow were assumed to be algal blooms based on extensive field observations (in GNP and elsewhere) by the sampling team. This targeted sampling approach is biased in that it focuses on specific patches of snow (in this case, orange, red, and green) and ignores snow algae that may be present on snowpack but not visible by eye (i.e. at low cell abundance) or algae that can be other colors including gray (Ling & Seppelt, 1998).

To reduce the impact of underlying substrata on spectral albedo, data collection and snow algae sampling were conducted primarily in areas with a snowpack thickness of at least 30 cm. Control sites with snow free of visible algae (hereafter referred to as *clean snow*) were sampled at multiple locations across both sections of the snowpack to account for site‐specific variability in other factors that might affect snow properties (e.g. inorganic deposition).

### Measurements of snow physical properties and spectral reflectance

In the field, and adjacent to each measurement site, snowpack depth was recorded using an avalanche probe, snow surface temperature was measured with a digital infrared thermometer, and snow water content was assessed using the SLF Snow Sensor (WSL, Switzerland). These measurements were taken just before the spectral reflectance measurements.

Before excavation of snow samples for laboratory analysis, spectral reflectance (as hemispherical‐directional reflectance factor, HDRF) of these sites was measured using an Analytical Spectral Devices (ASD) FieldSpec® 4 hyperspectral spectroradiometer (Malvern Panalytical, USA). Although the instrument measures wavelengths from 350 to 2500 nm, this study focuses on the spectral range of 400–1300 nm, chosen for its biological relevance in capturing the light absorption and reflectance properties of pigments, while avoiding unnecessary extension into the far‐infrared region where pigment signals are minimal (e.g. Di Mauro *et al*., 2024). Measurements were taken with a contact sensor probe that ensures consistent directional readings under uniform light intensity (halogen bulb, 2900 K color temperature), reducing contributions from stray light or temporal shifts in solar radiation. Before each set (i.e. color group) of snow measurements, a white reference measurement was obtained using a Spectralon panel.

To determine HDRF, surface reflectance was measured in triplicate for each of seven sampling sites for each color group (i.e. red, orange, and green) of snow algae (*n* = 21). We also measured the HDRF of *clean snow* (*n* = 6), to capture the variability in snowfield reflectivity outside of the red, orange, and green snow algae blooms. Reflectance (*R*) was determined by averaging *R* over two specified intervals: the visible range (400–700 nm) and the near‐infrared range (700–1300 nm).

To assess the potential utility of our data for remote‐sensing analysis, hyperspectral HDRFs were convolved with the spectral response of Sentinel 2A. Chlorophyll absorption was measured as the scaled area integral of Band 4 (665 nm) relative to Bands 3 (560 nm) and 5 (705 nm), following previously established methods (Painter *et al*., 2001; Gray *et al*., 2020; Khan *et al*., 2021), using the equation:

(Eqn 1) $$\text{RcontB}4=\mathrm{RB}3+\frac{\left(664-560\right)\left(\mathrm{RB}5-\mathrm{RB}3\right)}{\left(704-560\right)}$$

where RcontB4 is the spectral reflectance of the continuum between Bands 3 and 5, and RB3 and RB5 denote the spectral reflectance measured in Bands 3 and 5. The scaled integral of the band depth, IB4, was then calculated as

(Eqn 2) $$\mathrm{IB}4=\frac{\text{RcontB}4-\mathrm{RB}4}{\text{RcontB}4}$$

where RB4 is the reflectance measured in Band 4.

### Sample collection and biomass and inorganic particle estimations

A 2.5‐cm diameter corer was used to obtain surface snow samples from the top 5 cm of each site where reflectance measurements were conducted. The snow was placed in a sterile plastic bag and transferred to the laboratory within the next 2–3 h of collection. After melting the sample at room temperature, a 20 μl aliquot was taken for cell counts and the remaining volume was filtered onto ashed (24 h at 400°C) 0.7‐μm pore size Whatman GF/C filters, wrapped in aluminum foil and frozen (−20°C) until processing. Cell counts were conducted the same day of sampling by performing 9–16 replicate counts of a haemocytometer chamber (Hausser Scientific, Horsham, PA, USA) and a light microscope (Leitz LaborLux S, with ×10 objective). Photos of randomly selected cells were taken, and their lengths and widths were measured using ImageJ (v.1.52p; National Institutes of Health, Bethesda, MD, USA). Cell volumes (μm^3^) were estimated assuming spherical shapes for red snow algae and prolate spheroid shapes for orange snow algae according to Hillebrand *et al*. (1999). For green snow algae, both spherical and prolate spheroid shapes were observed.

Light‐absorbing inorganic particles (LAIPs) were assessed using loss on ignition with air‐dried sediments collected on pre‐weighed glass fiber filters. Half of each Whatman GF/C filter was placed in a 35‐ml porcelain crucible, dried at 60°C in a convection oven for 48 h, weighed, and then heated in a furnace at 400°C for 24 h. The difference in mass of the crucible and sediment before and after heating was assumed to represent the organic matter (OM) mass. The inorganic content (LAIPs) was estimated as the difference between the total mass and the OM mass.

### DNA extraction

Observations of cell shape, size, and general morphology during cell counts indicated that each replicate from each bloom color was composed of similar cells. Based on these observations, we assumed each replicate had a similar community composition and chose a single replicate (randomly) for DNA extraction and 18S rRNA amplicon sequencing. DNA was extracted from the filter using a DNeasy PowerSoil Kit (Qiagen) according to the manufacturer's instructions and Almela & Hamilton (2025). Two negative controls were included: an extraction blank to check for kit contamination and a field blank. The field blank was an ashed 0.7‐μm pore size Whatman GF/C filter washed with ultrapure (18.2 MΩ) water, and processed using the same equipment and techniques described. The concentration of DNA was determined using a Qubit DNA Assay kit (Molecular Probes, Eugene, OR, USA) and a Qubit 3.0 Fluorometer (Life Technologies, Carlsbad, CA, USA). No DNA was detected in the negative controls.

### 18S rRNA amplicon sequencing and analyses

DNA was submitted to the University of Minnesota Genomics Center (UMGC) for sequencing using a Nextera XT workflow, 2 × 300bp chemistry, and the primers 1391f and EukBr that target the V9 region of 18S SSU rRNA (Amaral‐Zettler *et al*., 2009; Stoeck *et al*., 2010).

Diversity and composition were assessed with Qiime v.2‐2024.10 (Bolyen *et al*., 2019). Briefly, cleaned and trimmed paired reads were filtered and denoized using the Dada2 plug‐in (Callahan *et al*., 2016). For chimera identification, 340 000 training sequences were used. Identified operational taxonomic units (OTUs), defined at 97% similarity, were aligned using Mafft (Katoh *et al*., 2002) and further processed to construct a phylogeny with fasttree2 (Price *et al*., 2010). Taxonomy was assigned to OTUs using the q2‐feature‐classifier (Bokulich *et al*., 2018) and blasted against the Silva v.138 99% 18S sequence database (Quast *et al*., 2012). All non‐Chlorophyta sequences were removed from the dataset. We then manually verified all sequences initially classified as Chlorophyta in the SILVA database using Blastn to confirm or refine their taxonomic assignments. For each query, we retained the consensus sequence with the highest score, ensuring 100% query coverage and > 90% sequence identity. When the top BLAST hit corresponded to an ‘uncultured eukaryote’ or ‘unknown Chlorophyta’, we selected the next best alternative hit based on the highest maximum score to improve the ecological interpretability of our dataset.

### Pigment extraction and analysis

To characterize and quantify the major pigments in algal cells, half of each filter with algal biomass was placed in sterile 15‐ml conical tubes, and 5 ml of a 7 : 2 acetone : methanol solvent was added. Cell disruption was performed by sonication (50% amplitude, 2 min). Samples were stored at −20°C overnight and then sonicated again (50% amplitude, 1 min) and centrifuged (5000 ***g***, 4°C, 10 min). An aliquot of the supernatant was filtered through a Millex® PVDF syringe filter (0.22 μm pore size). The filtered extracts were stored in glass vials at −80°C until further analysis.

To quantify pigment concentrations, 10 μl of total extract was analyzed with an Agilent 1100 series HPLC using a Discovery® 250 × 4.6 mm C18 column (Sigma‐Aldrich) with an isocratic mobile phase consisting of 50 : 20 : 30 methanol : acetonitrile : ethyl acetate flowing at 1 ml·min^−1^. Pigments were identified based on retention time and spectral matching to authentic standards of astaxanthin (3.17 min, 480 nm), Chl*b* (3.91 min, 649 nm), Chl*a* (4.39 min, 662 nm), and β‐carotene (6.90 min, 454 nm). Diagnostic wavelengths for Chl*a* and Chl*b* were chosen to prevent interference with astaxanthin. The amount of compound in each peak was determined by integrating peak areas using OpenLab software (Agilent) and comparing values to those from standard curves prepared for each pigment.

Pigment measurements were calculated in volumetric units of melted snow and hence vary with the depth and volume of snow excavated from the site. From the absolute concentrations of the analyzed pigments, we calculated the pigment signature as the percentage contribution of each pigment to the total pigment composition.

### Instantaneous radiative forcing (IRF)

For comparison with previous studies, we calculated snow algal IRF values over the photosynthetically active radiation (PAR) (400–700 nm) and NIR (700–1300 nm) regions following the methods used by Ganey *et al*. (2017). IRF (W m^−2^) was calculated based on the difference in reflectance between clean snow and algae‐covered snow, and considering the solar irradiance. To estimate the reflectance of clean snow, the six clean snow sample measurements were averaged using the arithmetic mean. The incoming spectral irradiance (W m^−2^ λ_400–700_ and λ_700–1300_) was obtained from the PVSystems solar irradiance program (https://pvlighthouse.com.au, last access: January 2025) for the time and location of sampling. IRF was estimated as follows (Ganey *et al*., 2017):

(Eqn 3) $$\mathrm{IRF}\;\approx \;\mathrm{Ed}\;\left(\lambda \right)\times \left(R_{\text{clean}}\;\left(\lambda \right)–R_{\text{algae}}\;\left(\lambda \right)\right)$$

where Ed is the incoming spectral irradiance (W m^−2^), and *R* is the reflectance (HDRF) for the clean or algae‐covered snow. Additionally, the data were presented relative to the algae biomass to standardize the measurements and facilitate comparison across the snow algae samples.

### Data analysis and statistics

All data analysis was performed in GraphPad Prism (v.8.0.1). To test significant differences between algal color groups, we performed a Kruskal–Wallis test, followed by Dunn's multiple comparison test to evaluate differences in their means. We used Pearson correlations to assess relationships between biological and physical parameters of snow. IRF was correlated with algal total biovolume, calculated as the product of cell volume (estimated from shape) and total cell density.

## Results

The three snow algae blooms occurred in a patchy distribution across the snowfield. Multiple individual patches of red, green, and orange snow were present, but no single patch exceeded 400 cm^2^ in area. In terms of vertical distribution, the orange bloom was predominantly concentrated near the snow surface, while the red and green blooms were more evenly distributed throughout the vertical snow profile, reaching ~2–3 cm (Supporting Information Fig. S1).

### Snow characterization

Water content and temperature were generally similar across the three sites. The red bloom samples (SA‐R) had an average surface temperature of 0.2 ± 0.1°C and a water content of 10.3 ± 1.3%. Orange samples (SA‐O) had an average surface temperature of 0.1 ± 0.0°C and a water content of 10.1 ± 0.3%. By contrast, green bloom samples (SA‐G) had the lowest water content (8.9 ± 0.6%) and surface temperature (−0.4 ± 1.1°C). Snow depth was significantly different between SA‐R (77 ± 7.2 cm) and both SA‐O (45.9 ± 11.4 cm) and SA‐G (39.3 ± 12.4 cm) (Table S1). For the clean snow, the average surface temperature was 0.1 ± 0.0°C, a snow depth of 70 ± 49.8 cm, and a water content of 10.4 ± 2.3%. Inorganic particle concentrations (LAIPs) on the snow varied across bloom patches, ranging from 1336.0 ± 1291.6 mg m^−2^ in SA‐R, to 3033.3 ± 3264.3 mg m^−2^ in SA‐G, and up to 8249.8 ± 5872.7 mg m^−2^ in SA‐O. A statistically significant difference was observed between LAIPs on red and orange bloom patches (*P* < 0.05) (Table S1).

### Algal distribution, abundance, and community composition

Red, orange, and green samples contained cells with distinct colors, morphologies, and sizes. Microscopy showed that red and orange snow were each dominated by a single cell type, while green snow had two dominant cell types. The following observations were noted for each bloom color (Table S2):

1. Red snow: Red or deep red, spherical cells with thick cell walls at an average density of 3175 ± 2290 cells·ml^−1^ (Fig. 1e). Chloroplasts were located centrally within the cells. The mean diameter of these cells was 35.96 ± 4.9 μm (*n* = 20). No other cell types were observed in the red snow samples.
2. Orange snow: Orange, oval‐shaped cells with flanged cell walls at a density of 19 154 ± 13 921 cells·ml^−1^ (Fig. 1g). The cells measured 25.9 ± 8.1 μm along the major axis and 14.6 ± 2.6 μm along the minor axis (*n* = 20). No other cell types were observed in the orange snow samples.
3. Green snow: (a) Green, ellipsoidal cells with smooth cell walls at an average density of 97 815 ± 2304 cells·ml^−1^ (Fig. 1f). These cells were 21.1 ± 5.2 μm along the major axis and 14.7 ± 7.1 μm along the minor axis (*n* = 20). The green ellipsoidal cells accounted for 97.8% of the total cells in the green snow samples. (b) Small, green, spherical cells at an average density of 2185 ± 2300 cells·ml^−1^ (Fig. 1f). These cells had a diameter of 8.3 ± 1.3 μm (*n* = 15). The green spherical cells accounted for 2.2% of the total cells in the green snow samples.

Despite the differences in cell density among colors, variations in cell size resulted in a similar total biomass per volume of melted snow across blooms (73.26 × 10^6^ vs 32.87 × 10^6^ μm^3^·ml^−1^ for red and green blooms, respectively). No flagellated algae were observed in any of the samples collected. No algae were observed in clean snow samples when examined under the microscope.

**Fig. 2:**
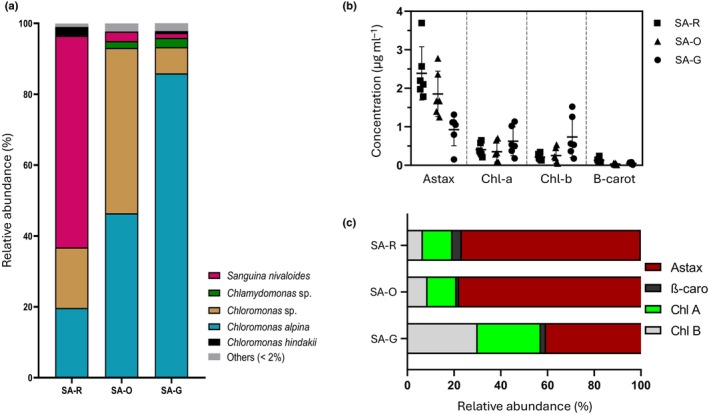
Overview of taxonomic and pigment composition across snow algae bloom colors. (a) Algal community composition of red (SA‐R), green (SA‐G), and orange (SA‐O) snow algae blooms based on 18S rRNA gene metabarcoding analysis. Concentration of the pigments all‐*trans*‐astaxanthin (Astax), Chl*a*, Chl*b*, and β‐carotene (β‐caro) in melted snow (b), along with their relative proportions (c) for each snow algae bloom color. Error bars represent the SD of the mean, illustrating the variability within the samples.

Based on 18S rRNA amplicons, distinct taxa were abundant in red, orange, or green blooms. The most abundant sequence in red snow (SA‐R) matched *Sanguina nivaloides* and accounted for 60% of total algal reads (Fig. 2a; Table S3). In green snow (SA‐G), the most abundant sequence matched *Chloromonas alpina* (86%). Sequences matching *Chloromonas* sp. and *Chloromonas alpina* represented 47% and 46% of total algal reads in orange snow (SA‐O), respectively. Only one cell type was observed in the orange bloom. Together, these three species accounted for > 94% of the total algal sequences across the three studied blooms, albeit in different proportions.

### Pigment composition and signature

A significant positive correlation was observed between total pigment content and algal abundance in SA‐R, SA‐O, and SA‐G, considering both cell density (*R* = 0.82, 0.86, and 0.90, respectively) and total biovolume (*R* = 0.83, 0.86, and 0.90, respectively). The pigment composition varied among samples in both absolute and relative values (Fig. 2b,c; Table S2). All‐*trans*‐astaxanthin was the predominant pigment in all three samples, with the highest concentration in SA‐R (2.39 ± 0.69 μg·ml^−1^), followed by SA‐O (1.85 ± 0.59 μg·ml^−1^) and SA‐G (0.92 ± 0.41 μg·ml^−1^). SA‐G exhibited threefold higher total Chl (Chl*a* and Chl*b*) concentrations compared to SA‐R and SA‐O (0.62, 0.60, and 1.4 μg·ml^−1^ in SA‐R, SA‐O, and SA‐G, respectively), whereas ß‐carotene was three to four times more abundant in SA‐R than in SA‐O and SA‐G (0.13 ± 0.06, 0.03 ± 0.02, and 0.05 ± 0.03 μg·ml^−1^ in SA‐R, SA‐O, and SA‐G, respectively).

Pigment profiles in the red and orange blooms were more similar to each other than to the green bloom (Fig. 2c). The combination of all‐trans astaxanthin and ß‐carotene pigments constituted 80.7% (±3.3%) of total pigments in SA‐R and 78.9% (±9.8) in SA‐O, but only 42.08% (±10.8) of total pigments in SA‐G. By contrast, Chls were relatively more abundant in SA‐G at 57.9% (±10.8) than in SA‐R (19.3% ± 3.3) and SA‐O (21.1% ± 9.8). These pigment abundances differed significantly (two‐way ANOVA, *P* < 0.01). The astaxanthin : Chl*a* ratio (Table S2) differed among bloom colors. The red bloom showed consistently high ratios (6.30 ± 1.64), reflecting a greater relative investment in astaxanthin. The green bloom had much lower ratios (1.58 ± 0.63), consistent with reduced carotenoid content relative to Chl*a*. The orange bloom exhibited the highest variability (8.46 ± 6.09), spanning values comparable to both red and green communities.

### Snow algae reflectance measurements

**Fig. 3:**
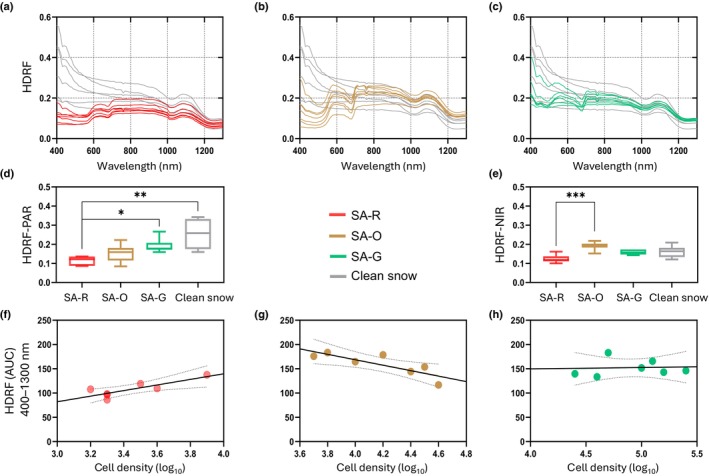
Spectral characterization of snow algae bloom reflectance across colors. (a–c) Spectral reflectance of red (SA‐R), green (SA‐G), and orange (SA‐O) snow algae blooms, along with clean snow as a reference, measured at Clements Mountain, GNP. Mean reflectance values are shown for (d) the photosynthetically active radiation (PAR, 400–700 nm) range and (e) the near‐infrared (NIR, 700–1300 nm) range. (f–h) Correlations between full‐spectrum reflectance (400–1300 nm) and log‐transformed cell density, with point colors indicating bloom color and replicate samples. Asterisks (*) indicate significant differences between color groups (*, *P* < 0.05; **, *P* < 0.01; ***, *P* < 0.001), as determined by a Kruskal–Wallis test followed by Dunn's multiple comparison test. Error bars represent the SD of the mean, illustrating the variability within the samples.

Spectral reflectance patterns reflected the variations in algal and snow composition across sites (Fig. 3a–c). In all cases, the presence of algae caused a reduction in reflectance within the visible range, with distinct dips in reflectance aligning with the peak absorption regions of astaxanthin and chlorophylls. In the algae sites, the red blooms (SA‐R) exhibited the lowest spectral reflectance while the green bloom (SA‐G) was the highest (Table S4). As expected, visibly clean snow had higher reflectance than the algae‐containing samples, as well as high variability, especially in the visible range.

Reflectance, averaged over the visible wavelength range (PAR), decreased by 55.5% in SA‐R (0.11 ± 0.02) compared to the visibly clean snow (0.25 ± 0.08), while SA‐G (0.19 ± 0.04) and SA‐O (0.15 ± 0.05) exhibited lower reductions in reflectance values (24.6% and 40.8%, respectively) (Fig. 3d). The differences in reflectance between red and clean snow and red and green snow were significant (*P* < 0.05). In the NIR wavelength range (Fig. 3e), red snow algae (0.12 ± 0.02) reflected on average 23.5% less energy than clean snow (0.16 ± 0.03), while green algae (0.16 ± 0.01) showed a reduction of 2.3%. Statistically significant differences were found only between red and orange snow patches (0.19 ± 0.02, *P* < 0.05), with the latter reflecting less energy than clean snow.

The correlation between cell density and reflectance measured in the field varied among samples. In SA‐R samples, reflectance was positively correlated to cell density (*r* ^2^ = 0.70; *P = 0.02*), indicating that reflectance increased with higher cell abundances (Fig. 3f). By contrast, reflectance was negatively correlated to cell density in SA‐O samples (*r* ^2^ = 0.68; *P = 0.02*), where increased algal presence corresponded to reduced reflectance (higher absorptance) (Fig. 3g). The correlation between cell density and reflectance in SA‐G samples was positive but not statistically significant (*r* ^2^ = 0.02; *P* > 0.05) (Fig. 3h). Similar patterns emerged when using total algal biovolume instead of cell density, with significant positive and negative relationships for red and orange blooms, respectively, and a weak, non‐significant trend for green blooms.

### Influence of snow algae blooms on radiative forcing

**Fig. 4:**
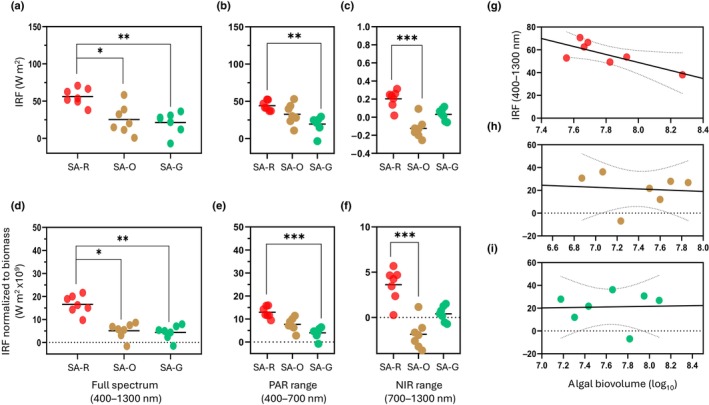
Instantaneous radiative forcing (IRF) of red (SA‐R), green (SA‐G), and orange (SA‐O) snow algae blooms calculated from the reflectance difference between clean and algae‐covered snow, accounting for solar irradiance. Panels show IRF across (a) the full spectrum (400–1300 nm), (b) the photosynthetically active radiation (PAR, 400–700 nm) region, and (c) the near‐infrared (NIR, 700–1300 nm) region. (d–f) IRF values normalized to biomass are also presented for the same spectral ranges. (g–i) Relationship between full‐spectrum IRF (400–1300 nm) and algal biovolume (log‐transformed) across red, orange, and green bloom colors. Asterisks (*) indicate statistically significant differences between color groups (*, *P* < 0.05; **, *P* < 0.01; ***, *P* < 0.001), as determined by Kruskal–Wallis test followed by Dunn's multiple comparison test.

The IRF integrated across the full spectrum (Fig. 4a; Table S4) was highest in red blooms (56.06 ± 11.25 W m^−2^). IRF in red blooms was significantly higher than both orange (25.21 ± 19.37, *P* < 0.05) and green blooms (21.33 ± 14.55, *P* < 0.05). Compared to red blooms, IRF decreased by *c*. 55% in orange and *c*. 62% in green blooms, indicating a reduced capacity of these communities to introduce radiant energy into the snowpack. Partitioning this effect by spectral region revealed that in the visible range, red blooms again dominated (44.04 ± 6.56 W m^−2^), while orange (32.69 ± 14.04) and green blooms (19.40 ± 11.05) showed progressively lower values. The red visible range IRF was significantly greater than green (*P <* 0.05). In the NIR range, IRF was positive for red blooms (12.05 ± 5.82 W m^−2^) but was near zero or negative for green (1.83 ± 3.93) and orange blooms (−7.48 ± 6.56). In the NIR range, red bloom IRF was significantly greater than orange (*P* < 0.05) (Fig. 4b,c).

When normalizing to algal biomass (cell density), IRF across the full spectrum showed the highest values in red blooms (16.54 ± 4.02 W m^−2^). IRF for red blooms was significantly higher for orange (5.49 ± 4.13) and green (4.38 ± 3.15) (Fig. 4d). Relative to red, IRF decreased by *c*. 65% in orange and *c*. 74% in green blooms. Partitioning by spectral range revealed that in the PAR, green blooms exhibited the lowest biomass‐specific IRF (2.63 ± 1.53), while red (5.66 ± 0.94) and orange (4.23 ± 1.67) were markedly higher (Fig. 4e), corresponding to increases of *c*. 216% and *c*. 161% compared to green. In the NIR (Fig. 4f), biomass‐specific IRF remained positive in red blooms (1.56 ± 0.77) but was near zero or negative in green (0.26 ± 0.55) and orange (−1.00 ± 0.86), with significant differences between red and green in the PAR and between red and orange in the NIR (*P* < 0.05). Normalizing by total algal biovolume yielded comparable results, with red bloom maintaining significantly higher IRF than orange and green.

Finally, correlation analysis between IRF and total biovolume showed a significant relationship only for red blooms (*r* ^2^ = 0.59, *P* < 0.05), whereas no significant correlations were found for orange or green blooms, reinforcing the dominant role of red blooms in driving snow darkening (Fig. 4g).

### Potential implications for remote sensing

Building on the distinct pigment signatures described above, we next examined Chl*a* content normalized by biomass to evaluate how these differences might impact remote‐sensing indices. When expressed per cell, red blooms contained 13.68 × 10^−5^ μg Chl*a* per cell, *c*. 23 times higher than green (0.60 × 10^−5^) and 8.4 times higher than orange blooms (1.62 × 10^−5^). By contrast, when normalized to total biovolume, green blooms showed the greatest Chl*a* content (1.86 × 10^−8^ μg per μm^3^), exceeding red (0.59 × 10^−8^) and orange blooms (0.56 × 10^−8^) by factors of *c*. 3.1 and *c*. 3.3, respectively. IB4 indices (Table S4), however, did not follow the same pattern: values were lowest in red blooms (2.42 ± 1.11) and substantially higher in orange (22.64 ± 4.83) and green blooms (19.26 ± 8.17). Significant positive correlations (*P* < 0.05) between IB4 and Chl*a* were found only in orange and green blooms (*r* ^2^ = 0.81 and 0.80 for cell density; *r* ^2^ = 0.78 and 0.82 for total biovolume).

## Discussion

### Snow algae blooms of different colors impact reflectivity in different ways

The presence of algae on snow altered its optical properties in the visible part of the spectrum. Snow algae blooms can appear in a range of colors due to differences in their pigment composition, which influences the energy balance of snow and accelerates melting rates (Dial *et al*., 2018). In our study, the spectral reflectance of red snow (SA‐R) was consistently lower than for green snow (SA‐G), while orange snow (SA‐O) fell in between the two. Compared to visibly clean snow, red snow blooms reduced reflectance by *c*. 55% across the 400–700 nm spectral range, while green snow caused a 25% reduction, showing a significant difference and a stronger impact of algae that appear red due to high levels of astaxanthin. NIR reflectance (700–1300 nm) was comparable between red and green blooms but markedly higher in the orange bloom. Previous studies have reported minimal impact on NIR albedo by snow algae because their pigments predominantly absorb within the visible or PAR range (Khan *et al*., 2021; Healy & Khan, 2023). SA‐O had a significantly higher inorganic content than SA‐R and SA‐G, but mineral dust also has a limited influence on NIR reflectance (Khan *et al*., 2021). Therefore, unmeasured physical properties of the snow, such as grain size or stratification, may be responsible for the higher NIR reflectance in orange snow.

To contextualize our findings, we compared our results to previous studies from different geographical locations. Lutz *et al*. (2014) estimated a snow albedo reduction of 26% for red blooms, and 31% for green blooms, on snow on the Greenland ice sheet. In the South Shetland Islands (Antarctica), Khan *et al*. (2021) estimated a contribution of 20% to the reduction of snow reflectivity for red blooms and 41% for green blooms. Other studies, which report data for either red or green blooms, estimated a snow albedo reduction of 13–17% for red blooms (Thomas & Duval, 1995; Lutz *et al*., 2016; Ganey *et al*., 2017) and *c*. 20% for green blooms (Gray *et al*., 2020). While the significance of snow water content, grain size, and other physical factors in influencing snow albedo and algal growth is well recognized (Lutz *et al*., 2014; Ganey *et al*., 2017; Gray *et al*., 2020; Khan *et al*., 2021), direct measurements of these specific parameters have not been reported in these studies, which present challenges for direct comparison.

Excavation depths is another complicating factor in making comparisons across studies. Published studies typically sample from a range of depths between 5 and 10 cm. The distribution of algae at different snow depths is not yet well documented, and pigment concentrations per cell can vary with depth (Khan *et al*., 2021). While it is known that subsurface algae effectively absorb light (Almela *et al*., 2025), the full extent of this phenomenon remains unknown. Regardless, even without accounting for the depth of the algal layer and the depth of excavation, our results suggest a more significant decrease in reflectance in red blooms, aligning with a 40% albedo reduction observed in the Alps due to the combined effect of red snow algae and mineral dust (Di Mauro *et al*., 2024).

The amount of inorganic particles did not differ significantly between clean snow and snow algae patches, although red and orange blooms showed significant differences from each other. Because even ‘clean’ snow contains LAIPs, the reflectance reduction measured in snow algae bloom samples can be largely attributed to the presence of algae and the physical properties of the snow. It is important to note that while our measurements focused on a highly localized area of a snowfield, previous studies (e.g. Gray *et al*., 2020; Khan *et al*., 2021) have integrated reflectance over a larger spatial scale. Our findings likely represent a higher estimate of reduction in reflectance by snow algae compared to albedo reduction across an entire snowfield due to the patchiness of snow algae distribution. Still, blooms reduce albedo and this impact could become more widespread under favorable conditions, although the specific environmental thresholds that promote such optimal development require further investigation.

Although red blooms absorbed more energy overall than green blooms, their spectral behavior differed around specific wavelengths. In particular, HDRF near 680 nm, the characteristic Chl*a* absorption band (Painter *et al*., 2001), was notably lower in the green snow, suggesting stronger pigment absorption relative to red blooms at this wavelength. To further interpret these patterns, we calculated the IB4, a remote‐sensing index derived from field‐measured HDRFs that is widely used to detect snow algae in satellite imagery. Consistent with previous observations, IB4 values were higher in green snow (5.47–27.72) than in red snow (1.57–4.49), indicating that the spectral properties measured in the field align with differences detectable at broader spatial scales. These findings align with Gray *et al*. (2020) who reported that absorbance by secondary carotenoids in red algae reduces reflectance in Band 3, masking Chl absorbance features in Sentinel‐2 bands and making automatic detection of red blooms challenging. These results indicate that IB4 is a reliable proxy for Chl*a* content in green snow algal blooms but fails to capture pigment dynamics in red blooms. Orange blooms contained astaxanthin levels similar to those found in red and approximately twice those in green snow blooms. However, IB4 values for the orange bloom (17.37–29.37), were on average higher than those for the green snow samples. Orange blooms were characterized by high astaxanthin content but consistently lower Chl*a* concentrations than red blooms. However, their IB4 values were higher than those of green snow and substantially greater than those of red blooms, suggesting that yet‐unresolved factors, possibly related to pigment organization or composition, may contribute to their distinct spectral behavior. Consequently, semi‐automated detection methods using IB4 can reliably identify astaxanthin‐rich orange blooms, whereas for red blooms, this index alone is insufficient and requires additional spectral information for accurate characterization.

### Snow colors represented by distinct community and pigment compositions

In this study, we compare three snow algae blooms of distinct colors and their impact on the energy balance of an alpine snowfield. 18S rRNA amplicons indicated a clear taxonomic distinction between bloom colors, with *Sanguina nivaloides* prevailing in red snow and *Chloromonas alpina* in green snow. The most abundant OTU in the orange bloom aligned with *Chloromonas* sp., while *Chloromonas alpina* was also detected at a similar relative abundance. *Sanguina nivaloides* has previously been associated with red blooms (Procházková *et al*., 2019a), whereas orange blooms have been linked to various *Chloromonas* species, including *C. krienitzii* (Procházková *et al*., 2020) and *C. hindakii* (Procházková *et al*., 2019b). In our dataset, however, the dominant sequence in orange blooms could not be resolved to the species level within *Chloromonas*, although *C. hindakii* was detected in the community at very low relative abundance. *Chloromonas alpina* has been recovered from green snow on glacial surfaces (Lutz *et al*., 2017). Despite recovering both *Chloromonas* sp. and *Chloromonas alpina* from orange snow samples, microscopy observations consistently revealed only a single cell type, which was orange in color and morphologically distinct from green snow algal cells. This discrepancy may reflect residual environmental DNA from earlier stages, or the presence of additional morphotypes not detected microscopically. Identifications are also based on partial 18S sequences – a larger fraction of the 18S rRNA gene or ITS markers might result in more robust taxonomic assignment. Our samples were collected at a single time point and thus we cannot resolve whether the recovery of distinct taxa reflects successional stages within the snowpack or independent communities. Nonetheless, the different snow colors corresponded to distinct algal compositions based on 18S rRNA amplicon sequencing.

The recovery of distinct snow algal communities from red, orange, and green blooms within the same snowpatch offers not only an ideal setting to quantify their differential contributions to albedo reduction, but also an opportunity to investigate why snow algae are predominantly red.

Members of the *Chlamydomonadaceae* have evolved complex life cycles that enable them to persist through winter and recolonize melting snow (Hoham & Duval, 2001; Soto *et al*., 2023), with motility sensitive to temperature and light, facilitating their re‐emergence after overwinter burial (Rea & Dial, 2024; Détain *et al*., 2025). Although insights from field observations and laboratory cultures have advanced our understanding of these perennial communities, their life cycles and ecology are not yet fully resolved (e.g. Matsumoto *et al*., 2024). Key stages remain difficult to observe directly due to the challenges of cultivation and the difficulty of detecting cells in deeper snow layers during early spring. Current hypotheses therefore integrate field and culture evidence, suggesting that cell stage is generally linked to the timing of exposure: early in the season, motile green flagellates inhabit deeper, shaded snow layers, whereas late‐season surfaces are dominated by orange‐ to red‐pigmented cysts (Williams *et al*., 2003; Remias *et al*., 2005; Hoham *et al*., 2006). Polar ecosystems, however, deviate from this pattern, as surface green blooms of *Chloromonas* are common even in highly exposed sites (Davey *et al*., 2019), whereas such occurrences are rarely reported in non‐polar regions (Procházková *et al*., 2023). Therefore, it remains uncertain whether pigment differences primarily reflect cell‐stage progression, or species‐specific ecological strategies.

Astaxanthin, a secondary ketocarotenoid typically synthesized under stress conditions such as nutrient depletion and intense solar irradiance (Bidigare *et al*., 1993; Remias *et al*., 2005; Leya *et al*., 2009; Sattler *et al*., 2012), was detected across all bloom colors, confirming that alpine snow algae experience sustained high‐light exposure. Its dominance in red (76.7%) and orange (77.8%) blooms, and lower abundance in green snow (40.6%), is consistent with visible bloom coloration, and provides further evidence of pigment differentiation among taxa. To better capture these differences, we examined the ratio of astaxanthin to Chl*a* as an indicator of the balance between photoprotection and photosynthetic capacity. The high astaxanthin : Chl*a* ratios observed in red (6.3) and orange (8.5) blooms compared to green snow (1.6) suggest contrasting strategies for balancing these functions. Elevated carotenoid‐to‐Chl ratios can reflect a reduction in chloroplast, and consequently thylakoid membrane, abundance (Procházková *et al*., 2024), which may limit photosynthetic capacity but optimize energy use under stress (Remias *et al*., 2005). Previously reported astaxanthin : Chl*a* mass ratios for snow algae (5.4 ± 4.5 across red, green, and orange samples) differ from our findings. For red blooms, reported ratios range widely, from 56 in Halbach *et al*. (2025) to 34 in Müller *et al*. (1998) for cells from Svalbard, and 3 for samples from Mt. Tateyama in Japan (Nakashima *et al*., 2021). For orange blooms, Procházková *et al*. (2019 b, 2024) reported ratios between 0.01 and 0.04 in Central Europe. These differences highlight the metabolic plasticity of snow algae, reflecting both life‐cycle transitions and taxonomic variability, as well as the influence of local environmental conditions on pigment composition.

Collectively, the contrasting pigment pools and relative abundances of dominant species support niche differentiation within the snowpack. Such patterns may reflect species‐specific adaptations or distinct life‐cycle stages co‐occurring within the same snowfield. The spatial segregation of bloom colors suggests that microhabitat heterogeneity shapes community composition and enables coexistence, while overall community functioning likely remains conserved across blooms despite shifts in taxonomic composition (Soto *et al*., 2022). These findings, together with the pigment complexity reported in other alpine and polar systems (Davey *et al*., 2019; Halbach *et al*., 2025), emphasize that snow algae blooms' coloration encodes both ecological and physiological information. Understanding how pigment composition mediates microhabitat occupation and vertical positioning within the snowpack, which may fluctuate over a 24‐h period (Ono & Takeuchi, 2025), remains an essential step toward linking cellular traits to large‐scale cryospheric processes.

### Differences in radiative forcing among snow algae bloom colors

**Fig. 5:**
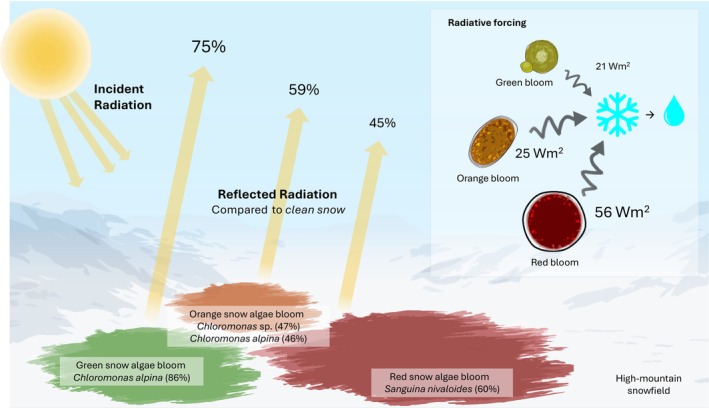
Summary of reflected radiation relative to clean snow (%) and instantaneous radiative forcing (W m^−2^) across snow algae blooms of different colors. The dominant taxon for each bloom color, according to the predominant 18S rRNA sequences obtained from those samples, and its relative abundance within the algal community, is shown.

Red snow algae patches exhibited both the highest light absorption in the visible and near‐infrared spectrum, and the greatest radiative forcing, with an average IRF that was *c*. 62% higher than that of the green bloom (Fig. 5). A significant correlation between IRF and algal biovolume was observed only in red blooms further supporting their dominant role in snow darkening, as increasing biomass directly enhances energy absorption. Although both green and red blooms occur in polar and mountainous regions, red blooms are consistently reported as prevalent and widespread (e.g. Healy & Khan, 2023). This widespread distribution indicates that their overall impact on snow albedo is likely much greater than that of other color groups, making them a key driver in albedo feedback in these ecosystems (Hotaling *et al*., 2021). These findings are in contrast with studies from Antarctica (Gray *et al*., 2020; Khan *et al*., 2021), where green snow patches occur more frequently and have a more significant impact on absorptance and IRF than red patches. These studies, along with others (e.g. Lutz *et al*., 2014), associate green algae with higher snow water content, which itself significantly reduces albedo (Thomas & Duval, 1995). By contrast, our results show minimal variability in snow surface properties among the samples, suggesting that differences in snow reflectivity across studies comparing green and red blooms may be influenced by varying snow conditions, which could obscure the pigment‐driven effects of algae. However, we measured snow surface temperature and water content during peak solar radiation hours, but did not assess how these parameters vary at other times of day or during the melting season, which may influence the observed values. Therefore, further research is needed to investigate diurnal variations in snow physical properties at the surface, as well as their changes in the vertical profile in relation to the various algae pigments.

Astaxanthin, the most abundant pigment in snow algae, occurs in various molecular forms, often bound to fatty acids and glucose, and accumulates in cytosolic lipid droplets that occupy a significant portion of the cell volume, fully surrounding the chloroplast (Ezzedine *et al*., 2023). Beyond coloration, it functions as an energy reserve (Remias *et al*., 2005) and cryoprotectant by displacing water within cells (Bidigare *et al*., 1993), while also shielding thylakoid membranes from intense visible and UVA radiation to reduce photoinhibition (Remias & Lütz, 2007; Ezzedine *et al*., 2023). Its consistently high abundance across all bloom colors, and especially in red and orange blooms, suggests that the pigment plays additional ecological roles beyond photoprotection (Ganey *et al*., 2017). Although astaxanthin may aid the overwinter survival of cyst‐like stages (Řezanka *et al*., 2013), its elevated concentration in non‐cyst orange cells indicates an active role during the melt season, promoting continued growth and the predominance of reddish populations (Hoham & Remias, 2020). This interpretation aligns with experimental evidence that red pigmentation enhances light absorption and heat generation in snow algae (Dial *et al*., 2018), providing a thermal advantage in extreme environments where maintaining optimal cellular function is critical. Accordingly, the disproportionate astaxanthin content in red and orange blooms is best understood as a strategy to accelerate snowmelt and secure access to liquid water in an otherwise frozen, water‐limited environment (Ganey *et al*., 2017). Conversely, the association of green algae with wet snow – previously reported but not detected in our study – suggests that their distribution is governed primarily by water availability rather than light stress, since astaxanthin was still the predominant pigment. This interpretation is consistent with observations from Antarctic snowfields, where *Chloromonas*‐dominated green communities often develop on part of the snowfields where meltwater accumulates (e.g. Gray *et al*., 2020). It may also apply to our green bloom samples, which were collected from areas with lower snow depth that likely experienced greater melting and transient liquid water availability, even if surface water was not detected due to rapid percolation through the coarse snow matrix (Kohshima *et al*., 1993).

Radiative forcing values indicated that algae with higher astaxanthin content, predominantly red and orange cells, have the greatest potential for absorbing and re‐emitting energy, especially within the visible spectra. This process not only influences algal physiology but also impacts the surrounding environment, as heat absorption and reemission by pigmented cells contribute to localized melting of adjacent ice crystals (Dial *et al*., 2018). However, our results suggest that it is not only astaxanthin concentration that determines its impact on the snow surface's energy balance. Correlation analyses between algal biomass – measured as biovolume to account for variations in cell size and shape – and HDRF in the field revealed that biomass influenced reflectance in a color‐specific manner. Red blooms exhibited a positive relationship, with higher biomass linked to increased overall reflectance, likely reflecting enhanced light scattering associated with their larger size. By contrast, orange blooms, despite having astaxanthin concentrations comparable to red blooms, showed the opposite trend, with biomass correlating to greater apparent energy absorption, yet this effect did not translate into higher IRF, possibly because much of the absorbed energy is retained within cells rather than *trans*ferred to the surrounding snow matrix. These significant patterns (*r* ^2^ ≈ 0.7, *P* < 0.05) appear robust, but should still be interpreted cautiously given the limited sample size.

Recent findings suggest that the ability of snow algae to modify snow reflectance results from an interplay of pigments, cell density, and morphological traits such as size and shape, collectively described as the Effective Albedo Reduction Surface (Almela *et al*., 2024). While pigments remain central to sunlight absorption and heat dissipation, the optical effect is further modulated by how these pigments are organized within the cell and by cell geometry itself. In particular, larger cells can enhance both scattering and internal absorption, amplifying their radiative impact even when pigment concentrations are modest. Thus, pigment content alone does not determine reflectance, but acts in concert with cell structure to shape the overall optical response of the bloom. Ezzedine *et al*. (2023), using Chl autofluorescence, found that orange *Sanguina* cells displayed higher fluorescence intensity than red cells, suggesting differences in intracellular pigment organization. The packaging of pigments can significantly influence the reflective properties of algae. A comparative study on snow and ice algae by Halbach *et al*. (2025) showed that the ‘pigment packaging effect’ is especially pronounced in snow algae, with its impact on cellular energy absorption influenced by factors such as the presence of pigment‐protein complexes, cell size and internal structure. One possible explanation for the lower radiative forcing of orange vs red algae in our study could lie in this difference. However, the molecular forms of astaxanthin in snow algae have not been analyzed, and further research is required. Additionally, other pigments not quantified in this study (e.g. lutein) may also be contributing to these differences. These findings highlight the complex interactions between pigmentation, pigment packaging, energy balance, and environmental feedback in snow ecosystems, emphasizing the challenges of studying and predicting their response to environmental changes.

## Competing interests

None declared.

## Author contributions

PA and TLH planned and designed the research. PA and TLH conducted fieldwork. AZ and TN performed the pigment analysis. PA conducted all the other analyses and analyzed the data. PA wrote the initial manuscript. PA, TLH and JJE contributed to interpreting the data and revised the final version of the manuscript. All authors approved the submitted version.

## Disclaimer

The New Phytologist Foundation remains neutral with regard to jurisdictional claims in maps and in any institutional affiliations.

## Supporting information


**Fig. S1** Representative snow cores from red, orange, and green snow algae blooms.
**Table S1** Summary of sample characteristics for red, orange, and green snow algae blooms.
**Table S2** Summary of cell abundance, algal biovolume, and pigment composition of snow algae blooms.
**Table S3** Relative abundance and taxonomic identification of snow algae across bloom colors.
**Table S4** Reflectance properties and radiative forcing of snow algae blooms.
Please note: Wiley is not responsible for the content or functionality of any Supporting Information supplied by the authors. Any queries (other than missing material) should be directed to the *New Phytologist* Central Office.

## Data Availability

Sequence Accessions – sequence data is archived at the SequenceRead Archive (SRA) at NCBI under the accessions: BioProject (PRJNA1244840) and BioSamples (SAMN47732474–SAMN47732476).
