# Supplementary material for: Community‐driven variations in snow algae color modulate snow albedo reduction
Source: New Phytol. 2025 Nov 25;249(4):1739–52. doi: 10.1111/nph.70775 (PMC12825401; doi:10.1111/nph.70775)
Supplement: Supplementary file 1 — Fig. S1 Representative snow cores from red, orange, and green snow algae blooms. Table S1 Summary of sample characteristics for red, orange, and green snow algae blooms. Table S2 Summary of cell abundance, algal biovolume, and pigment composition of snow algae blooms. Table S3 Relative abundance and taxonomic identification of snow algae across bloom colors. Table S4 Reflectance properties and radiative forcing of snow algae blooms. Please note: Wiley is not responsible for the content or functionality of any Supporting Information supplied by the authors. Any queries (other than missing material) should be directed to the New Phytologist Central Office. [file NPH-249-1739-s001.docx]

## *New Phytologist* Supporting Information

Article title: Community-driven variations in snow algae color modulate snow albedo reduction

Authors: Almela, Pablo; Elser, James J.; Zmuda, Anthony; Niehaus, Thomas; Hamilton, Trinity L.

Article acceptance date: 31 October 2025

The following Supporting Information is available for this article:

**Fig. S1** **Representative snow cores from red, orange, and green snow algae blooms.** Representative snow cores (2.5 cm diameter, 5 cm depth) collected from red (SA-R), orange (SA-O), and green (SA-G) snow algae blooms, showing characteristic color differences and pigment distribution within the upper snow layer..

**
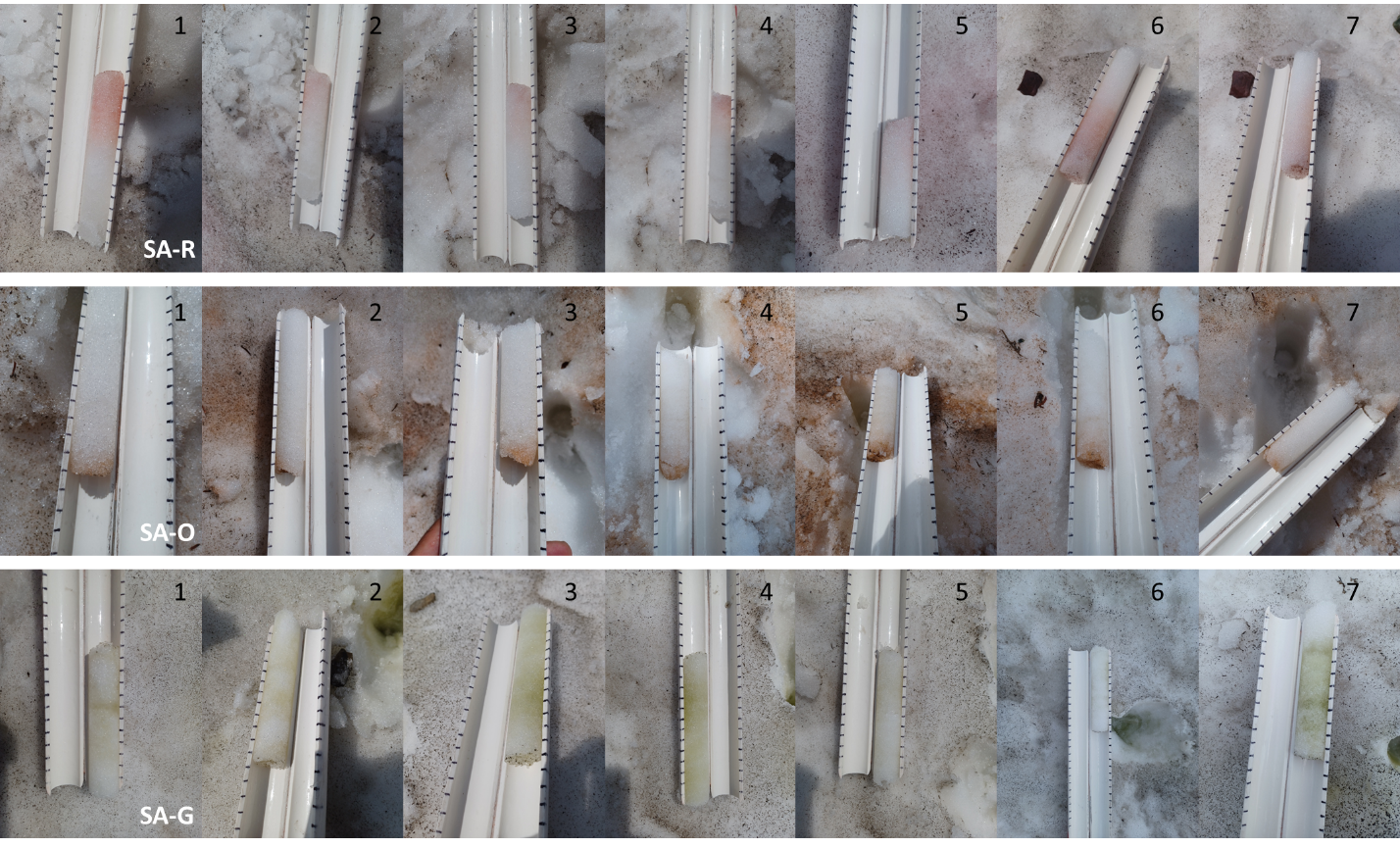
**

**Table S1 Summary of sample characteristics for red, orange, and green snow algae blooms.**

Summary of sample characteristics for red (SA-R), orange (SA-O), and green (SA-G) snow algae bloom samples, including GPS coordinates, elevation (m a.s.l.), snow depth (cm), water content (%), temperature (°C) at the time of sampling, and inorganic matter content (%). NA indicates samples used for sequencing analyses.

| Sample | GPS, elevation | Elevation | Aspect | Depth (cm) | Water content (%) | Temperature (°C) | IM (mg/m^2^) | IM (%) |
| --- | --- | --- | --- | --- | --- | --- | --- | --- |
| SA-R | 48°41'35" N 113°44'6" W | 2215 m | -3 |  |  |  |  |  |
| 1 |  |  |  | 74 | 9.4 | 0.3 | 0.0 | 34 |
| 2 |  |  |  | 74 | 10.6 | 0.3 | 0.0 | 0 |
| 3 |  |  |  | 74 | 10.3 | 0.3 | NA | NA |
| 4 |  |  |  | 74 | 11.6 | 0.2 | 1189.4 | 30 |
| 5 |  |  |  | 74 | 10.7 | 0.2 | 2411.4 | 36 |
| 6 |  |  |  | 74 | 8.0 | 0.1 | 3226.1 | 24 |
| 7 |  |  |  | 93 | 11.6 | 0.1 | 1189.4 | 31 |
| SA-O | 48°41'47"N 113°44'13"W | 2121 m | -19 |  |  |  |  |  |
| 1 |  |  |  | 34 | 10.1 | 0.1 | 2818.7 | 94 |
| 2 |  |  |  | 33 | 10.2 | 0.1 | NA | NA |
| 3 |  |  |  | 38 | 10.0 | 0.1 | 6077.4 | 18 |
| 4 |  |  |  | 64 | 9.9 | 0.1 | 8521.4 | 61 |
| 5 |  |  |  | 52 | 10.3 | 0.2 | 18704.7 | 55 |
| 6 |  |  |  | 52 | 10.7 | 0.1 | 10150.7 | 61 |
| 7 |  |  |  | 48 | 9.6 | 0.1 | 3226.1 | 71 |
| SA-G | 48°41'35.2"N 113°44'05.7"W | 2212 m | -3 |  |  |  |  |  |
| 1 |  |  |  | 35 | 9.3 | -2.1 | 0.0 | 0 |
| 2 |  |  |  | 35 | 9.1 | -2.0 | 2818.7 | 16 |
| 3 |  |  |  | 35 | 8.8 | 0.3 | 1596.7 | 53 |
| 4 |  |  |  | 45 | 8.9 | 0.3 | 8114.1 | 51 |
| 5 |  |  |  | 45 | 9.8 | 0.3 | 5670.1 | 32 |
| 6 |  |  |  | 20 | 8.1 | 0.3 | 0.0 | 0 |
| 7 |  |  |  | 60 | 8.1 | 0.2 | NA | NA |
| Snow | 48°41'47.8"N 113°44'12.1"W | 2214 m | -3 |  |  |  |  |  |
| 1 |  |  |  | 45 | 11.1 | 0.1 | 4530.5 | 62 |
| 2 |  |  |  | 45 | 11.4 | 0.1 | 9508.1 | 75 |
| 3 |  |  |  | 45 | 11.3 | 0.1 | 12286.2 | 83 |
| 4 |  |  |  | 73 | 12.3 | 0.0 | missing | missing |
| Snow | 48°41'46"N 113°44'13"W | 2230 m | -19 |  |  |  |  |  |
| 5 |  |  |  | 43 | 5.8 | missing | missing | missing |
| 6 |  |  |  | 178 | 10.5 | missing | missing | missing |

**Table S2** **Summary of cell abundance, algal biovolume, and pigment composition of snow algae blooms**

Summary of cell abundance, algal biovolume, pigment concentrations, and astaxanthin:Chl-a ratios for red (SA-R), orange (SA-O), and green (SA-G) snow algae bloom samples. Pigment concentrations (astaxanthin, Chl-a, Chl-b, and β-carotene) are expressed in μg·mL⁻¹. NA indicates samples used for sequencing analyses.

| Sample | cells density  (mL-1) | biovolume  (μm3) | Astax  (μg·mL-1) | Chl-a  (μg·mL-1) | Chl B  (μg·mL-1) | β-caro  (μg·mL-1) | Astax:Chl-a |
| --- | --- | --- | --- | --- | --- | --- | --- |
| SA-R.1 | 8111.11 | 187175904.37 | 3.70 | 0.65 | 0.34 | 0.24 | 5.69 |
| SA-R.2 | 2888.89 | 66665390.60 | 1.78 | 0.29 | 0.15 | 0.09 | 6.26 |
| SA-R.3 | 1888.89 | 43588909.24 | NA | NA | NA | NA | NA |
| SA-R.4 | 2111.11 | 48717016.21 | 2.10 | 0.38 | 0.20 | 0.13 | 5.54 |
| SA-R.5 | 1555.56 | 35896748.78 | 1.97 | 0.21 | 0.13 | 0.08 | 9.29 |
| SA-R.6 | 3666.67 | 84613764.99 | 2.57 | 0.58 | 0.29 | 0.15 | 4.44 |
| SA-R.7 | 2000.00 | 46152962.72 | 2.20 | 0.34 | 0.17 | 0.09 | 6.56 |
| SA-O.1 | 7000.00 | 20168017.98 | 1.40 | 0.08 | 0.04 | 0.01 | 18.22 |
| SA-O.2 | 9444.44 | 27210817.91 | NA | NA | NA | NA | NA |
| SA-O.3 | 5222.22 | 15045981.67 | 1.25 | 0.09 | 0.06 | 0.01 | 13.87 |
| SA-O.4 | 22777.78 | 65626090.25 | 1.67 | 0.32 | 0.23 | 0.03 | 5.20 |
| SA-O.5 | 42857.14 | 123477661.10 | 2.77 | 0.70 | 0.53 | 0.05 | 3.97 |
| SA-O.6 | 31000.00 | 89315508.20 | 2.36 | 0.65 | 0.45 | 0.06 | 3.65 |
| SA-O.7 | 15777.78 | 45458072.27 | 1.67 | 0.28 | 0.19 | 0.02 | 5.86 |
| SA-G.1 | 123000.00 | 39808458.00 | 1.12 | 0.54 | 0.56 | 0.06 | 2.08 |
| SA-G.2 | 98000.00 | 31717308.00 | 1.11 | 0.50 | 0.53 | 0.04 | 2.23 |
| SA-G.3 | 154000.00 | 49841484.00 | 1.32 | 1.13 | 1.26 | 0.07 | 1.16 |
| SA-G.4 | 53000.00 | 17153238.00 | 0.80 | 0.37 | 0.36 | 0.03 | 2.13 |
| SA-G.5 | 224000.00 | 72496704.00 | 1.05 | 1.02 | 1.52 | 0.08 | 1.03 |
| SA-G.6 | 23000.00 | 7443858.00 | 0.15 | 0.18 | 0.17 | 0.01 | 0.85 |
| SA-G.7 | 36000.00 | 11651256.00 | NA | NA | NA | NA | NA |

**Table S3** **Relative abundance and taxonomic identification of snow algae across bloom colors.** Relative abundance and taxonomic identification of snow algae in the red bloom (SA-R), orange bloom (SA-O) and green bloom (SA-G). The table shows the proportion of each algal taxon in relation to the total algal community, along with corresponding BLASTN results used for taxonomic assignment, including Query Cover, Percent Identity (Per. Ident), and GenBank Accession number.

| **SA-G** | **SA-O** | **SA-R** | **Taxonomy** | **Query Cover** | **Per. Ident** | **Accession** |
| --- | --- | --- | --- | --- | --- | --- |
| 1.4 | 2.7 | 59.7 | *Sanguina nivaloides* | 100% | 99.38% | LC648245.1 |
| 85.9 | 46.4 | 19.7 | *Chloromonas alpina* | 100% | 99.38% | HQ404865.1 |
| 7.4 | 46.7 | 17.1 | *Chloromonas sp.* | 100% | 96.25% | LC648242.1 |
| 2.6 | 1.9 | 0.0 | *Chlamydomonas sp.* | 100% | 97.50% | FR865535.1 |
| 0.5 | 0.0 | 2.5 | *Chloromonas hindakii* | 100% | 95.32% | MN251865.1 |
| 0.0 | 1.7 | 0.0 | *Pseudochlorella signiensis* | 100% | 93.87% | LT560366.1 |
| 1.1 | 0.5 | 0.2 | unknown *Sanguina* | 100% | 98.16% | OR101571.1 |
| 1.1 | 0.2 | 0.8 | <1% |  |  |  |

**Table S4** **Reflectance properties and radiative forcing of snow algae blooms.** Reflectance, radiative forcing, and IB4 index values red (SA-R), orange (SA-O), and green (SA-G) snow algae bloom samples. Hemispherical–directional reflectance factor (HDRF) is expressed as the area under the curve (AUC) for the full spectrum (400–1300 nm), the photosynthetically active radiation (PAR, 400–700 nm), and the near-infrared (NIR, 700–1300 nm) regions. Instantaneous radiative forcing (IRF, W m⁻²) was estimated assuming an incoming solar irradiance of 337.4 W m⁻². The IB4 index was calculated as described in Equation 2 of the Methods section.

| Sample | HDRF-AUC  (400-1300) | HDRF-AUC  (400-700) | HDRF-AUC  (700-1300) | IRF  (W·m^-2^) | IB4 |
| --- | --- | --- | --- | --- | --- |
| SA-R.1 | 137.5 | 40.62 | 96.88 | 37.1 | 3.38 |
| SA-R.2 | 119 | 36.58 | 82.41 | 41.2 | 1.60 |
| SA-R.3 | 86.37 | 25.97 | 60.4 | 52.2 | 2.26 |
| SA-R.4 | 97.01 | 25.75 | 71.26 | 52.4 | 4.49 |
| SA-R.5 | 107.8 | 40.63 | 67.16 | 37.0 | 1.57 |
| SA-R.6 | 109.7 | 36.31 | 73.38 | 41.4 | 1.87 |
| SA-R.7 | 97.83 | 30.97 | 66.86 | 47.0 | 1.78 |
| SA-O.1 | 183.4 | 66.14 | 117.2 | 10.8 | 17.37 |
| SA-O.2 | 164.7 | 49.51 | 115.2 | 27.9 | 18.15 |
| SA-O.3 | 176 | 53.92 | 122 | 23.4 | 17.94 |
| SA-O.4 | 144.3 | 34.23 | 110.1 | 43.8 | 29.37 |
| SA-O.5 | 116.7 | 25.21 | 91.45 | 53.0 | 26.73 |
| SA-O.6 | 153.9 | 37.97 | 115.9 | 39.9 | 25.21 |
| SA-O.7 | 178.7 | 66.14 | 117.2 | 30.1 | 23.71 |
| SA-G.1 | 165.8 | 61.83 | 104 | 14.8 | 23.36 |
| SA-G.2 | 151.7 | 55.83 | 95.83 | 21.0 | 22.04 |
| SA-G.3 | 143.1 | 52.68 | 90.46 | 24.2 | 26.31 |
| SA-G.4 | 183.1 | 79.4 | 103.7 | -3.5 | 18.64 |
| SA-G.5 | 146.2 | 52.22 | 94.01 | 24.7 | 27.72 |
| SA-G.6 | 139.6 | 51.71 | 87.9 | 25.3 | 11.27 |
| SA-G.7 | 133.5 | 61.83 | 104 | 29.4 | 5.27 |
| Clean-1 | 175.7 | 72.31 | 103.4 | NA | NA |
| Clean-2 | 148.4 | 54.08 | 94.31 | NA | NA |
| Clean-3 | 120 | 47.73 | 72.3 | NA | NA |
| Clean-4 | 164.5 | 81.73 | 82.75 | NA | NA |
| Clean-5 | 223.9 | 98.4 | 125.5 | NA | NA |
| Clean-6 | 204.4 | 102.1 | 102.3 | NA | NA |
